# Prognostic nutritional index may not be a good prognostic indicator for acute myocardial infarction

**DOI:** 10.1038/s41598-019-51044-9

**Published:** 2019-10-11

**Authors:** Yisong Cheng, Hong Li, Dongze Li, Lianjing Liang, Yu Jia, Liqun Zou, Fanghui Li, Xingyu Zhu, Hong Qian, Na He, Zhi Zeng, Rui Zeng, Yu Cao, Zhi Wan

**Affiliations:** 1 Department of Emergency Medicine, Laboratory of Emergency Medicine, West China Hospital, Sichuan University, Chengdu, China; 2 Disaster Medicine Center, Sichuan University, Chengdu, China; 3 Department of Cardiac Surgery, West China Hospital, Sichuan University, Chengdu, China; 4 Department of Cardiology, West China Hospital, Sichuan University, Chengdu, China

**Keywords:** Prognostic markers, Myocardial infarction, Risk factors

## Abstract

The prognostic nutritional index (PNI) has been applied in acute myocardial infarction (AMI) recently.However, the application of PNI in AMI needs verification. This was a prospective cohort study. Patients diagnosed with AMI were enrolled. PNI was calculated as (serum albumin (SA in g/L)) + (5 × total lymphocyte count (TLC) × 10^9^/L). Modified PNI (mPNI) was analyzed by logistic regression analysis to reset the proportion of SA and TLC. The primary outcome was all-cause death. A total of 598 patients were enrolled; 73 patients died during follow-up. The coefficient of SA and TLC in the mPNI formula was approximately 2:1. The area under the receiver operating characteristic curve of SA, TLC, PNI, mPNI and GRACE in predicting death for patients with AMI was 0.718, 0.540, 0.636, 0.721 and 0.825, respectively. Net reclassification improvement (NRI) between PNI and mPNI was 0.230 (*p* < 0.001). Integrated discrimination improvement (IDI) was 0.042 (*p* = 0.001). Decision curve analysis revealed that mPNI had better prognostic value for patients with AMI than PNI; however, it was not superior to SA. Thus, PNI may not a reliable prognostic predictor of AMI; after resetting the formula, the value of PNI in predicting prognosis of AMI is almost entirely due to SA.

## Introduction

Ischemic cardiovascular disease is the most common cause of death, and its frequency is increasing worldwide. The annual incidence of hospital admission for acute myocardial infarction (AMI) varies between 90-312/100,000 per year in Europe^1^. Through the advent of modern antithrombotic therapy^2^, secondary prevention, and percutaneous coronary intervention (PCI)^3,4^, there has been a decrease in acute and long-term mortality due to cardiac causes; however, the one-year mortality due to ST segment elevation myocardial infarction (STEMI) is approximately 10%^5^. Thus, early risk stratification management has been utilized, and many risk predictive indicators have been applied to predict the short- and long-term prognosis of patients with AMI^6,7^.

The prognostic nutritional index (PNI) was first proposed by Buzby *et al*. to assess the prognosis of patients undergo gastrointestinal surgery in 1980^8^. Computer-based stepwise linear regression was conducted using serum albumin (SA), serum transferrin, triceps skinfold, and delayed hypersensitivity to reflect the baseline nutritional status. In 1984, Onodera simplified the linear predictive model using immune-nutritional indicators according to the following equation: PNI = SA (g/L) + (5 × total lymphocyte count [TLC] × 10^9^/L). This study showed that the model could be used safely when PNI was over 45^9^, but some reports showed that the optimal cut-off value of PNI is variable^10,11^. PNI was used to prognosticate various malignancies^12–16^, pulmonary embolism^17^, and other diseases. PNI proved to be an effective indicator for assessing nutritional and immunological conditions of patients with cancer and was shown to influence patient prognosis through the local immune response^16^.

PNI was mostly used to predict prognosis of cancer patients and has been applied in AMI in some recent studies^18,19^. Myocardial infarction is closely related to SA levels^20,21^ and to inflammation processes^22^. However, low SA levels in myocardial infarction may be due to its role as an antioxidant, anti-inflammatory, and antiplatelet aggregation agent, and not primarily due to its nutritional aspect^23–25^. The pathophysiological mechanisms of SA and TLC in cancer and AMI may not be the same. SA and TLC may have different roles when predicting prognosis for patients with cancer versus AMI, which means that the Onodera PNI calculation used for patients with cancer may not necessarily be applicable to patients with AMI. PNI may be valuable in evaluating the prognosis of AMI patients, however, the application of PNI in assessing prognosis of AMI patients has not been verified and neither compared with traditional risk score such as Global Registry of Acute Coronary Events (GRACE) in these studies^18,19^. The aims of this study were 1) to investigate whether Onodera’s PNI calculation is applicable to patients with AMI and 2) to adjust the PNI formula and determine if it improves the prognosticating value of PNI for AMI, and compared with traditional GRACE risk score.

## Results

### Baseline characteristics

This study enrolled 626 patients with AMI; 28 were excluded based on the exclusion criteria. Thus, 598 patients were analyzed (95.5%). The mean age was 64 ± 13 years, and 456 (76.4%) were male patients. On prospective follow-up, 73 patients died at a median of 14.8 (9.3–17.8) months. Baseline characteristics are shown in Table 1. The mean value of continuous PNI was 47.8 ± 6.0, and 182 patients (30.4%) had a PNI score of 1. Dead patients were older, had a higher rate PNI of 1, had higher heart rates, Killip class, D-Dimer, creatinine, interleukin 6, C-reaction protein, NT-proBNP, troponin and GRACE score, and had lower body mass index, SA levels, and left ventricular ejection fraction (LVEF) than those survived.

**Table 1:** Demographic variables and baseline clinical characteristics.

Variable	Survival(n = 525)	Death(n = 73)	*p* Value
**Demographics**			
Age, years	63.4 ± 13.1	73.0 ± 10.3	<0.001
BMI, kg/m^2^	24.3 ± 5.7	22.2 ± 3.8	0.002
Males, n (%)	405(77.1)	51(69.9)	0.162
Smoking, n (%)	290(55.2)	38(52.1)	0.670
SBP, mmHg	125.3 ± 23.5	122.9 ± 26.2	0.430
DBP, mmHg	77.8 ± 15.8	76.2 ± 18.1	0.433
HR, /min	79.0 ± 17.5	90.6 ± 22.4	<0.001
Killip class ≥2, n(%)	189(36)	56(76.7)	<0.001
**Laboratory findings**			
WBC, ×10^9^/L	10.1 ± 3.5	10.8 ± 4.6	0.19
TLC, ×10^9^/L	1.3(1.0–1.7)	1.2(0.8–1.8)	0.247
Platelet, ×10^12^/L	174.5 ± 74.0	164.2 ± 61.9	0.258
D-dimer, mg/L	0.4(0.2–0.9)	1.1(0.5–2.8)	<0.001
Creatinine, μmol/L	77(65–91)	93(70–132)	<0.001
TG, mmol/L	1.5(1.0–2.4)	1.1(0.8–1.6)	<0.001
TC, mmol/L	4.6 ± 1.1	4.2 ± 1.4	0.026
HDL, mmol/L	1.2 ± 0.3	1.2 ± 0.4	0.257
LDL, mmol/L	2.8 ± 0.9	2.6 ± 1.2	0.051
Serum albumin, g/L	41.0 ± 4.0	37.7 ± 5.3	<0.001
Proteinuria, n(%)	138(26.3)	19(26.0)	0.348
IL-6,pg/ml	9.3(5.6–21.1)	17.0(7.9–55.2)	0.003
CRP, mg/L	4.6(2.5–12.3)	8.5(4.2–25.7)	0.001
NT-proBNP, pg/ml	593(151–2042)	2225(532–6425)	<0.001
cTnT, ng/L	402(68–1468)	716(141–2543)	0.031
LVEF, %	55.5 ± 22.0	43.4 ± 12.4	<0.001
Gensini score	67.6 ± 44.5	71.2 ± 44.4	0.609
GRACE score	155.3 ± 36.6	208.1 ± 41.5	<0.001
PNI = 1, n(%)	142(27.0)	40(54.8)	0.001

BMI, body mass index; SBP, systolic blood pressure; DBP, diastolic blood pressure; HR, heart rate; WBC, white blood cell; TLC, total lymphocyte count; TG, triglyceride; TC, cholesterol; HDL, high-density lipoprotein; LDL, low-density lipoprotein; IL-6, interleukin 6; CRP, C-reactive protein; NT-BNP, N-terminal pro-brain natriuretic peptide; cTnT, troponin; LVEF, left ventricular ejection fraction; GRACE, Global Registry of Acute Coronary Events; PNI, prognostic nutritional index.

### mPNI formula

mPNI was calculated using logistic regression to reset the coefficient of SA and TLC for all-cause death. The equation used was as follows: Logit (P) = (5.142–0.184 × SA + 0.104 × TLC); the coefficient of SA and TLC in the mPNI formula was approximately 2:1. The conditional probability of mPNI in predicting all-cause death can also be calculated.

### mPNI and long-term mortality

**Figure 1:**
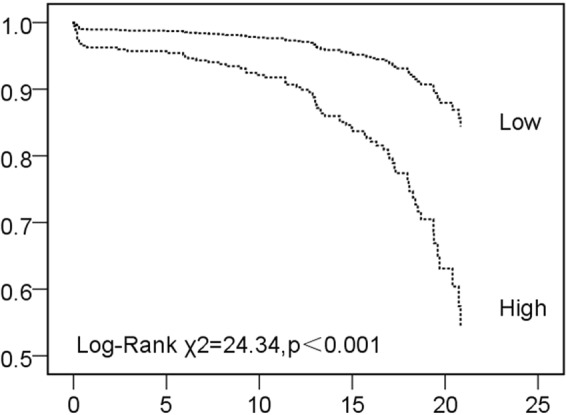
Kaplan-Meier survival analysis of high risk and low risk of mPNI. mPNI, modified prognostic nutritional index.

The conditional probability of mPNI was divided according to the average into low- and high-risk groups. The high-risk group showed significantly higher mortality than the low-risk group (19.1% vs. 5.4%, *p* < 0.001). Consistently, cumulative survival was significantly lower in the high-risk group than the low-risk group during follow-up (53.7% vs. 84.6%, *p* < 0.001) **(**Fig. 1**)**.

Univariate logistic regression indicated that PNI, mPNI, BMI, serum albumin, triglyceride, cholesterol, low-density lipoprotein, LVEF and GRACE were associated with all-cause death of AMI patients during follow-up **(**Table 2**)**. After adjusting for potential confounders in the multivariate logistic regression analysis, mPNI was still independently associated with all-cause death (mPNI high risk vs. low risk, OR = 2.595, 95% CI, 1.084–6.212, *p* = 0.032), as well as LVEF and GRACE. However, PNI was not an independent factor (PNI 1 vs. 0, OR = 0.535, 95% CI, 0.241–1.188, *p* = 0.124).

**Table 2:** Univariate and multivariate logistic regression analysis of all-cause death.

Variable	Univariate analysis			Multivariate analysis		
	HR	95%CI	*p*	HR	95%CI	*p*
BMI	0.832	0.769–0.899	<0.001	0.929	0.834–1.034	0.177
Serum albumin	0.854	0.812–0.898	<0.001	1.019	0.946–1.099	0.618
TG	0.994	0.990–0.998	<0.001	0.996	0.990–1.001	0.142
TC	0.994	0.988–0.999	0.037	1.013	0.985–1.042	0.355
LDL	0.763	0.583–0.998	0.048	0.596	0.184–1.927	0.387
LVEF	0.909	0.882–0.936	<0.001	0.933	0.904–0.964	<0.001
GRACE	1.036	1.028–1.044	<0.001	1.026	1.016–1.036	<0.001
PNI (1 vs. 0)	3.208	1.941–5.302	<0.001	0.535	0.241–1.188	0.124
mPNI (high vs. low)	4.052	2.265–7.249	<0.001	2.595	1.084–6.212	0.032

BMI, body mass index; TG, triglyceride; TC, cholesterol; LDL, low-density lipoprotein; LVEF, left ventricular ejection fraction; GRACE, Global Registry of Acute Coronary Events; PNI, prognostic nutritional index; mPNI, modified prognostic nutritional index.

### Calibration and discrimination abilities of PNI and mPNI

**Figure 2:**
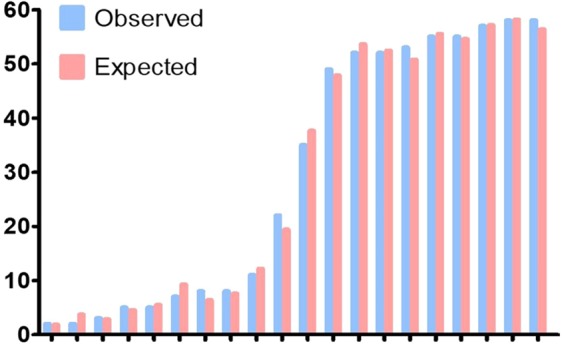
Calibration histogram of mPNI of predicting death based on logistic regression. mPNI, modified prognostic nutritional index.

After reset the formulation of SA and TLC, goodness of fit test showed mPNI was non-statistical significance using the Hosmer-Lemeshow test (*p* = 0.950), which means mPNI had a good goodness of fit. Calibration histogram also graphically showed the observed frequency is almost the same as the predicted frequency in each event risk groups **(**Fig. 2**)**.

**Figure 3:**
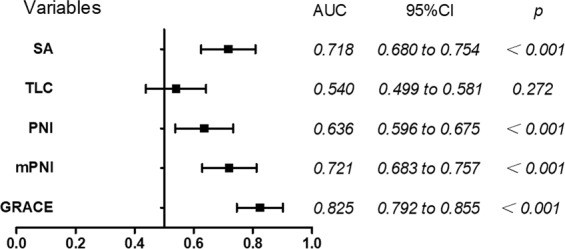
AUC of SA, TLC, PNI, mPNI and GRACE of predicting all-cause death. AUC, area under the receiver-operating characteristic curve; SA, serum albumin; TLC, total lymphocyte count; PNI, prognostic nutritional index; mPNI, modified prognostic nutritional index; GRACE, Global Registry of Acute Coronary Events.

The AUCs of SA and TLC in predicting death were 0.718 (95% CI, 0.680–0.754, *p* < 0.001) and 0.540 (95% CI, 0.499–0.581, *p* = 0.272), respectively. The AUC of mPNI in predicting death was significantly higher than that of PNI (0.721 vs. 0.638, *p* = 0.004), but less than that of GRACE score (0.825, 95% CI, 0.792–0.855, *p* < 0.001, Fig. 3), and the difference was significant (p = 0.002).

The risk discrimination ability of PNI and mPNI was compared by net reclassification improvement (NRI), the mortality of AMI patients in our study was 12.2%, thus we used 8% and 25% as the arbitrary thresholds to define low, intermediate and high risk. The category NRI of PNI and mPNI was 0.229 (95% CI, 0.093–0.366, *p* < 0.001), and IDI was 0.042 (95% CI, 0.017–0.068, *p* = 0.001). Seven patients in the death group were reclassified to the moderate or high-risk group and 70 patients in the survival group were reclassified to the low or moderate risk group. mPNI showed good prognostic performance and significant net reclassification than PNI **(**Table 3**)**. The NRI between SA and mPNI was no statistical significance, NRI less than 0.001 (95% CI, −0.062–0.063, *p* = 0.978,) and IDI was 0.009 (95% CI, −0.002–0.020, *p* = 0.106).

**Table 3:** Reclassification across pre-defined risk thresholds.

Death	mPNI			
PNI	<0.08	0.08–0.25	≥0.25	total
<0.08	5	4	0	9
0.08–0.25	7	35	11	53
≥0.25	0	1	10	11
total	12	40	21	73
				NRI+ = 0.096
**Survival**				
<0.08	107	39	0	145
0.08–0.25	124	221	18	363
≥0.25	0	3	13	16
total	231	263	31	525
				NRI− = 0.133

PNI, prognostic nutritional index; mPNI, modified prognostic nutritional index.

**Figure 4:**
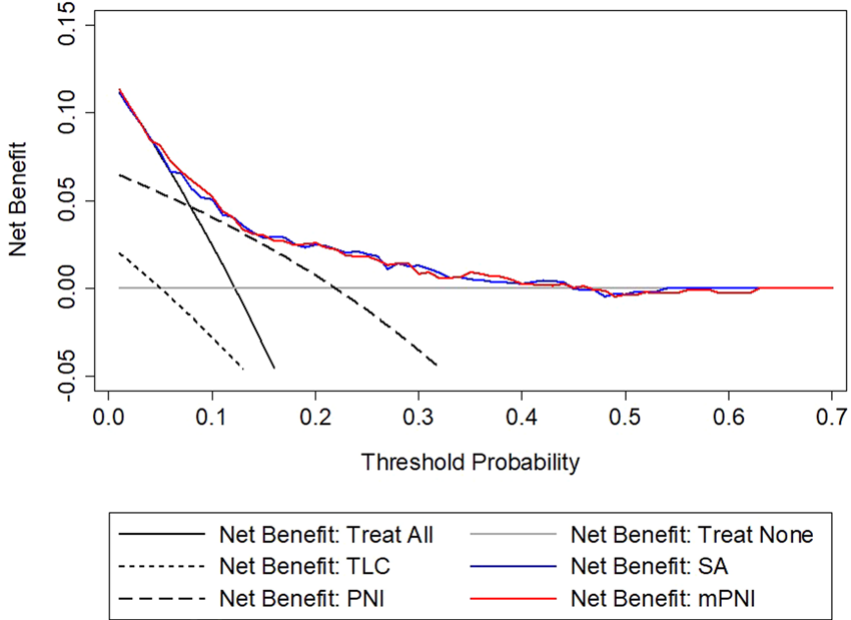
Decision curve analysis for SA, TLC, PNI and mPNI. SA, serum albumin; TLC, total lymphocyte count; PNI, prognostic nutritional index; mPNI, modified prognostic nutritional index.

Since the AUCs of SA and mPNI were close to each other, DCA was performed to compare the prognostic value of both. DCA showed that mPNI had better prognostic value for patients with AMI than PNI at any threshold probability; however, it was not superior to SA. TLC did not show any net benefit in the current study **(**Fig. 4**)**. The DCA graphically showed that mPNI had a higher predictive prognosis value of AMI patients than PNI, but almost same as SA.

## Discussion

In the present study, we investigated the prognostic value of PNI in patients with AMI and reset the PNI formula to see if this would improve its prognostic value in patients with AMI. Results showed that PNI may not be a reliable prognostic predictor of AMI. After modifying the PNI calculation, mPNI did not show better predictability than SA and was inferior to the traditional GRACE score. The value of PNI in predicting prognosis in patients with AMI comes almost entirely from SA. To the best of our knowledge, the present study is the first to reset the calculation and verify the prognostic value of PNI in patients with AMI.

PNI was proposed to evaluate surgery risk and prognosis of patients with gastrointestinal malignancy^9^. SA, synthesized in the liver, is the most abundant protein in circulation and is a good indicator reflecting the nutritional status of patients with cancer. Malnutrition is associated with increased morbidity and mortality in patients with cancer^26,27^. On the other hand, lymphocytes play an important role in eradicating the formation and progression of tumors^28^, and they can also eliminate cancer cells and inhibit cancer cell proliferation, invasion, and migration^29,30^. Thus, PNI is a significant prognostic factor in patients with cancer.

In recent studies, PNI was associated with prognosis in patients with STEMI^18,19^. However, these studies did not separately analyze the prognostic value of SA and TLC, neither did not reset the formula used for AMI, and neither compared with GRACE score. Hypoalbuminemia is an independent predictor of in-hospital and long-term adverse outcomes of AMI. SA is a negative indicator of inflammation, which means that its concentration decreases in the presence of inflammation^31^. It is also an abundant and important circulating antioxidant using ligand binding and free radical-trapping activities during AMI^23,32^. Lastly, it is a significant inhibitor of platelet activation and aggregation, which play an important role in the development of thrombosis in AMI^33^. Thus, the role of SA in AMI may due to its inflammation, antioxidant activity, and antiplatelet aggregation^24,34^. Lymphocyte count is an index of immunoreaction; a low lymphocyte count may be associated with a pre-existing immunosuppression process, which indicates an inadequate immunological reaction in cardiovascular diseases^35^. AMI patients with lymphocytopenia are more likely to develop endothelial dysfunction, platelet activation, and thrombogenesis^36,37^. Since the pathophysiology of cancer and AMI is not the same, we hypothesized that SA and TLC have different roles when predicting prognosis in patients with cancer versus AMI. The coefficient of SA and TLC in the mPNI formula was approximately 2:1 in our study, not 1:5 in Onodera PNI calculation, agreed to our hypothesis.

The present study showed that the AUC of SA in predicting death was higher than that of TLC and PNI, meaning that SA plays a vital role in the pathophysiologic mechanism of AMI. PNI showed weak prognostic value. After adjusting the formula of PNI, we found that mPNI had higher prognostic value than PNI but was not significantly superior to SA; this indicates that TLC may have a limited role in predicting the outcomes of patients with AMI. There was no significant difference in TLC between the survival group and death group in this study. TLC is an immune-inflammatory biomarker, and patients with AMI are more likely to develop lymphocytopenia due to increased inflammatory-related lymphocytes apoptosis. Decreasing lymphocyte count, and the smaller proportion of TLC in mPNI may partly explain that TLC does not play an important role in the current study. Besides, lymphocytopenia acts as a marker for ongoing nonspecific atherosclerotic inflammatory processes. AMI is an acute inflammatory, thrombotic disease and closed related to blood lipid, thus many studies emphasized that the early recognition and management is important to improve prognosis of patients with AMI^2,38,39^.

GRACE score is a traditional easy and cheap tool used for prognosis of AMI patients, our study indicated that the AUC of mPNI for predicting mortality of AMI patients was inferior to GRACE score. GRACE score has been recognized for its high efficiency and simplicity, but still need eight variables. The mPNI was not originally used for prognostic assessment of AMI, and only included two indicators, thus the results were acceptable.

The present study has several limitations. First, it is a single-center study and included a small sample size. Results in the present study need to be validated further in larger populations. Second, only the calculation of PNI was verified, and other inflammatory biomarkers to rebuild PNI were not analyzed. Third, only the admission PNI and mPNI were evaluated; it is unclear if subsequent changes in the values can provide additional prognostic value.

Our study showed that PNI may not be a reliable prognostic predictor of AMI. After adjusting the formula, mPNI had higher discrimination and calibration abilities than PNI, but was comparable to SA and inferior to GRACE score. The value of PNI in predicting prognosis in patients with AMI comes almost entirely from SA. TLC plays a small role in calculating PNI. This result may prompt us to investigate other biomarkers to rebuild PNI when applied to predict the prognosis of patients with AMI.

## Methods

### Study design

This was a single-center, prospective cohort study. The study adhered to the tenets of the Declaration of Helsinki; the Human Ethical Committee of West China Hospital of Sichuan University approved the study protocol. We obtained informed consent from all participants involved in the study.

### Study population

Patients diagnosed with AMI at the Emergency Department of West China Hospital of Sichuan University between October 2016 and September 2017 were enrolled in the current study. The diagnostic criteria for AMI was based on the fourth definition of myocardial infarction^40^: a rise and/or fall of troponin values with at least one value above the 99th percentile upper reference limit (URL) together with clinical evidence of acute myocardial ischemia (i.e., ischemic symptoms, electrocardiogram new ischemic changes or development of pathological Q waves, imaging evidence, angiography, or autopsy identification). Exclusion criteria included usage of antithrombotic drugs within 24 hours, lack of laboratory data, and patients with cancer, active or chronic infections diseases, end-stage renal disease, or liver failure.

### Data collection

Baseline and clinical data such as vital signs, medical history, past history, and laboratory data were obtained using standard case report forms on admission. An electrocardiogram was promptly obtained upon admission using an electrocardiograph (iMAC1200, Wuhan Zoncare Bio-Medical, Hubei, China). Current smokers were defined as those who had smoked at least 100 cigarettes during their lifetime and were still smoking within the previous 1 month^41^. Hypertension was diagnosed when the systolic or diastolic blood pressure was ≥140/90 mmHg using a mercury-column sphygmomanometer after 10 minutes of rest or when taking antihypertensive drugs. Diabetes mellitus was defined as a fasting glucose level of ≥7 mmol/L, hemoglobin A1C level of ≥6.5%, random venous blood glucose level of ≥11.1 mmol/L, or use of antidiabetic drugs. Venipuncture was completed at room temperature, and blood samples were filled in standard tubes and centrifuged rapidly. Hematology analytes, including hemoglobin, platelet count (PLT), white blood count (WBC), and TLC were analyzed using the automated hematology analysis system Beckman Coulter LH750 (Beckman Coulter Inc., Brea, CA, USA). SA, alanine aminotransferase (AST), aspartate aminotransferase (ALT), urea nitrogen, and creatinine levels were analyzed using the Architect c16000 analyzer (Abbott Diagnostics).

### PNI calculation

PNI was calculated according to the patients’ baseline clinical characteristics using the formula: SA (g/L) + (5 × TLC × 10^9^/L). Scores of ≥45 or <45 were assigned a PNI of 0 or 1, respectively.

### Endpoint and follow-up

Prospective clinical follow-up after discharge was accomplished by telephone or questionnaire forms; in-hospital data were reconfirmed using hospital records. Only patients who completed the follow-up were included in this study. The primary outcome was all-cause death.

### Statistical analysis

Continuous variables are expressed as mean ± standard deviation or median with interquartile range. Continuous variables were tested for normality distribution using the Kolmogorov-Smirnov test; normally distributed variables were evaluated using the independent sample t-test, and non-normally distributed variables were tested using the Mann-Whitney U-test. Categorical variables are presented as frequencies and percentages and were analyzed using the chi-square test or Fisher’s exact test. Variables showing *p* < 0.05 in univariate logistic regression analysis were selected in the multivariate model.

Modified prognostic nutritional index (mPNI) was calculated by logistic regression analysis to reset the coefficient of SA and TLC with all-cause death. The area under the curve (AUC) was analyzed to compare the prognostic value of AMI. A calibration histogram combined with the Hosmer-Lemeshow test was applied to evaluate the calibration of mPNI. Reclassification analysis is widely recommended for assessing the discrimination of risk prediction^42^, so net reclassification improvement (NRI) and integrated discrimination improvement (IDI) were applied to analyze the degree by which mPNI improved predictive ability as compared to PNI or SA. Vickers *et al*.^43^ suggested the use of decision curve analysis (DCA) to evaluate the diagnostic and prognostic models and to compare the prediction value of these models. Two tailed *p*-values of <0.05 were considered statistically significant. All analyses were performed using SPSS for Windows version 21.0 (SPSS Inc., Chicago, IL, USA) and Stata version 14.0 (StataCorp LP, College Station, TX).
